# Low oxygen environment effect on the tomato cell wall composition during the fruit ripening process

**DOI:** 10.1186/s12870-024-05226-x

**Published:** 2024-06-06

**Authors:** Agata Leszczuk, Nataliia Kutyrieva-Nowak, Artur Nowak, Artur Nosalewicz, Artur Zdunek

**Affiliations:** 1 Institute of Agrophysics, Polish Academy of Sciences, Doświadczalna 4, Lublin, 20-290 Poland; 2 Department of Industrial and Environmental Microbiology, Institute of Biological Sciences, Maria Curie- Skłodowska University, Akademicka 19, Lublin, 20-033 Poland

**Keywords:** Anoxia, Cell wall, Fruit, Hypoxia, Polysaccharides, Ripening process

## Abstract

**Background:**

Oxygen concentration is a key characteristic of the fruit storage environment determining shelf life and fruit quality. The aim of the work was to identify cell wall components that are related to the response to low oxygen conditions in fruit and to determine the effects of such conditions on the ripening process. Tomato (*Solanum lycopersicum)* fruits at different stages of the ripening process were stored in an anoxic and hypoxic environment, at 0% and 5% oxygen concentrations, respectively. We used comprehensive and comparative methods: from microscopic immunolabelling and estimation of enzymatic activities to detailed molecular approaches. Changes in the composition of extensin, arabinogalactan proteins, rhamnogalacturonan-I, low methyl-esterified homogalacturonan, and high methyl-esterified homogalacturonan were analysed.

**Results:**

In-depth molecular analyses showed that low oxygen stress affected the cell wall composition, i.e. changes in protein content, a significantly modified in situ distribution of low methyl-esterified homogalacturonan, appearance of callose deposits, disturbed native activities of β-1,3-glucanase, endo-β-1,4-glucanase, and guaiacol peroxidase (GPX), and disruptions in molecular parameters of single cell wall components. Taken together, the data obtained indicate that less significant changes were observed in fruit in the breaker stage than in the case of the red ripe stage. The first symptoms of changes were noted after 24 h, but only after 72 h, more crucial deviations were visible. The 5% oxygen concentration slows down the ripening process and 0% oxygen accelerates the changes taking place during ripening.

**Conclusions:**

The observed molecular reset occurring in tomato cell walls in hypoxic and anoxic conditions seems to be a result of regulatory and protective mechanisms modulating ripening processes.

**Supplementary Information:**

The online version contains supplementary material available at 10.1186/s12870-024-05226-x.

##  Introduction

In the face of climate change and population growth, an efficient chain of food supply is needed to ensure food security. Hypoxia is a stress factor for plant productivity in some world regions, and still little is known about the main pathway via which plants signal oxygen deficiency. The general outline of the responses of plant metabolism to a fall in internal oxygen concentrations is the subject of many reports [1, 2]. A decline in internal oxygen concentrations leads to adaptive responses that vary in their time scales and levels of control. Mechanisms that allow plants to avoid anoxia/hypoxia include a rapid decrease in metabolism to minimize oxygen consumption and morphological changes increasing oxygen entry. Hypoxia maintains the induction of genes encoding enzymes involved in anaerobic metabolism and induces genes involved in the amelioration of oxidative stress as well as growth and elongation-related genes [3].

The progress toward understanding the molecular basis of the response of horticultural crops to hypoxia has economic significance as well, i.e. it helps to optimize storage conditions and reduce losses during packaging and transport [4, 5]. Reduction in the concentration of oxygen and increasing the concentration of carbon dioxide in the atmosphere leads to a decrease in the intensity of respiration. It has been hypothesized that this physiological response plays a key role in extending the shelf life of detached plant organs. It also slows down the aging of vegetables or fruits, extending the postharvest storage of the product [6]. The optimal composition of the atmosphere for vegetables and fruits is usually 0–5% carbon dioxide and 1.5–3% oxygen. An oxygen concentration below 8% slows down the rate of ethylene production in cells. However, it should be underlined that an insufficient oxygen concentration (below 1%) endangers anaerobic fermentation, which causes tissue damage and accumulation of substances with an undesirable taste and smell and provokes the growth of anaerobic microorganisms deleterious to humans [6].

Moreover, it is also well known that a hypoxic atmosphere affects the rate of ripening [6]. At low oxygen stress, ethylene biosynthesis decreases, which is directly associated with delaying ripening and improper colour development [7]. Reduced levels of oxygen have been used for the extension of shelf life and control of postharvest pathogens and disinfestations. A low oxygen environment provokes detrimental effects, mainly abnormal ripening, flesh browning, and a large increase in ethanol and acetaldehyde [8–10]. Underlying this mechanism is the suppression of the synthesis of enzymes induced in the course of ripening and the activation of new enzymes. The concentration of O_2_ in the range from 2.5 to 5.5% stimulates the activity of cellulase and polygalacturonase and induces the appearance of new polypeptides involved in ripening [11]. Glucanases (β-1,3-glucanase, endo-β-1,4-glucanase) catalyze the hydrolysis of glycosidic bonds in polysaccharides and are involved in the reconstruction and exchange of cell wall components during many physiological processes in plants [12]. In turn, the physiological function of guaiacol peroxidase activity is related to biosynthetic processes and defense against abiotic and biotic stresses, i.a. production of reactive oxygen species as signaling mediators in the cell wall involved in defense mechanisms against pathogen infection [13]. Further, the appearance of enzymes and new constituents is directly related to cell wall properties and composition. During ripening, the cell wall disassembly is responsible for fruit softening and textural changes. As ripening progresses, the cell wall becomes increasingly hydrated as the pectin-rich middle lamella is partially hydrolysed and its continuity is broken. Changes in the cohesiveness of the pectin gel determine the separation between fruit cells, which in turn affects the structure of the whole ripe fruit organ [14, 15]. The damage to cellular membranes at tomato ripening results in loss of turgor and overall fruit softening. In turn, the absence of decreased membrane integrity during ripening and senescence of stored fruit and vegetables correspond with lipid peroxidation [16].

The link between stressful oxygen concentration and enzymatic activity justifies the study of the cell wall in the context of hypoxic conditions. The cell wall is the key building block of fruit tissue as it is extremely mechanically strong and associated with processes involved in the formation of the structure of fruits and changes occurring along with the progress of ripening and senescence [17–19]. Moreover, the cell wall is engaged in the cell response to stress factors appearing during storage [20]. Our previous data about alternations in the composition and spatio-teporal pattern of distribution of extensin, arabinogalactan proteins, rhamnogalacturonan-I, low methyl-esterified homogalacturonan, and high methyl-esterified homogalacturonan were strictly connected with modifications ongoing in fruit [19–21]. Moreover, changes in the presence or molecular parameters of one of them disrupted the presence of the others, which is closely related to their mutual interactions and the formation of assembly in an extracellular matrix. In the latest model of cell wall structure, APAP1 complex, it was assumed that the abovementioned components and their binding abilities influence the general properties of the cell wall [22, 23]. However, the cellular and molecular changes in fruit cell walls resulting from modified oxygen concentration conditions are not well understood. In this research, the response of model organism *Solanum lycopersicum* at two stages of ripening to 0% and 5% oxygen concentrations is evaluated. The aim of this work was to identify the components of the cell wall that are altered most substantially in response to low oxygen conditions. Constituents that are implicated in cell wall softening were examined with the use of microscopic and in vitro molecular and enzymatic assays. Tools based on antibodies that recognize specific epitopes corresponding to the individual components of cell walls were used as well. The results provided an answer to the question of whether low oxygen stress induces changes at the cellular level or exerts an effect on the ripening process. The in-depth molecular analyses showed that low oxygen stress almost immediately induced changes in the cell wall composition.

##  Material

Tomato *Solanum lycopersicum* fruits cv. ‘Moneymaker’ at two stages of the ripening process – at the start (breaker – BR) and at the last stage of the process (red ripe – RR) were used as research material. Tomato plants were grown at the glasshouse under standard conditions of temperatures at the range of 18–27 °C and relative humidity at the range of 60–80%. The material was collected from 10 tomato fruits from each experiment variant. Shortly after collection, the fruits were subjected to storage conditions differing in oxygen concentration in Gas Exchange Chamber 3010 GWK1 connected to Gas-Exchange System GFS-3000 (Heinz Walz GmbH, Effeltrich, Germany) for 24 h and 72 h in a low oxygen environment: 0% - anoxia and 5% - hypoxia at 22 °C air temperature and 60% relative air humidity. Also, the fruit collected at the BR and RR stages directly from the plant without storage were used in the experiment and named ‘0 h’. For the control experiment (21% oxygen - normoxia), the fruits were kept in the air for 24 h and 72 h. Representative fruit tissues were collected from different fruit samples, immediately frozen in liquid nitrogen, stored for further analysis at -80 °C, and/or fixed for the microscopic visualization. The methods were performed according to the procedure described in detail in our previous publication [24].

##  Methods

###  Microscopic studies on cell wall assembly: immunofluorescence labelling, toluidine blue & aniline blue staining

Commercial antibodies recognizing cell wall constituents were used for immunocytochemical studies (Kerafast, USA). The following antibodies were selected: antibodies recognizing extensin (LM1), arabinogalactan proteins (LM2), rhamnogalacturonan-I (LM16), low methyl-esterified homogalacturonan (LM19), and high methyl-esterified homogalacturonan (LM20).

The microscopic studies were performed according to our previous work [20]. Cube-shaped samples were obtained by excision of the external part of the fruit, which included the epidermal and hypodermal layers, and parenchyma from a depth of ca. 1 cm under the skin. Briefly, samples were fixed in 2.5% paraformaldehyde and 0.25% glutaraldehyde (Sigma Aldrich, USA) in 0.1 M PBS pH 7.4. The dehydratation procedure was carried out in a series of ethanol (10-99.8% ethanol) for 30 min at each concentration. Subsequently, the material was embedded in LR White resin (Sigma Aldrich, USA) and then polymerization was carried out in tightly closed gelatine capsules at 55ºC for 48 h. The fruit embedded in LR White resin were cut into 1-µm thick sections using an ultramicrotome (PowerTome XL, RMC Boeckeler, USA). The sections were stained with Aniline blue for callose detection (Sigma, USA). For immunocytochemical labelling, the sections were placed on poly-L-lysine coated glass slides (Sigma, USA), hydrated with distilled water, and treated with 1% bovine serum albumin (BSA, Sigma Aldrich) in 0.2 M PBS, pH 7.4, for 30 min. Next, the sections were incubated with a primary monoclonal antibody diluted 1:50 with 0.1% BSA in 0.2 M PBS, pH 7.4. Subsequently, the slides were washed with PBS and incubated with Alexa Fluor 488 antibody (Thermo Fisher Scientific, Denmark) diluted 1:200 with 0.1% BSA in 0.2. M PBS, pH 7.4. Finally, the sections were washed in PBS and distilled water. For better visualization, double-staining with Calcofluor White (Sigma Aldrich, USA) was carried out. The microphotographs were taken using an Olympus BX51 CLSM microscope with the corresponding software FluoView v. 5.0. (Olympus Corporation, Tokyo, Japan). All parameters (i.e. laser intensity, gain) were kept constant for all imaging. Figures were edited using the CorelDrawX6 graphics program.

###  Studies on enzymatic activity

####  Protein extraction

Three grams of tomato fruits were frozen in liquid nitrogen and ground into a fine powder in the mortar. Soluble proteins were extracted with 6 mL of 50 mM phosphate buffer (pH 7.5) containing 1mM EDTA (Sigma-Aldrich, USA), 1 mM PMSF (Sigma-Aldrich, USA), and 1% PVPP (Sigma-Aldrich, USA) for 1 min [25]. The suspension was filtered through a Miracloth filter (Merck Millipore, USA) and centrifuged (10.000 rpm) at 4 °C for 20 min. The samples were stored on ice between each step. The protein content of the extracts was determined using the Bradford method (Bradford reagent, Sigma-Aldrich, USA). The extracts obtained were used to determine the enzyme activity.

####  β-1,3-glucanase and endo-β-1,4-glucanase activity

The β-1,3-glucanase activity was determined by the release of glucose from laminarin from *Laminaria digitata* (Sigma-Aldrich, USA) [12]. The endo-β-1,4-glucanase activity was determined by the release of glucose from CMC – carboxymethylcellulose (Sigma-Aldrich, USA) [26]. 100 µL of 1% laminarin (β-1,3-glucanase) or 1% CMC (endo-β-1,4-glucanase) in acetate buffer (pH 5.6) was added to 50 µL of the extract and incubated for 2 h and 3 h repetitively at 37 °C with continuous stirring. Next, 100 µL of the mixture was transferred to 400 µL of water, and 1.5 mL of DNS reagent (0.53% 3,5-dinitrosalicylic acid (Sigma-Aldrich, USA), 0.99% NaOH, 0.415% NaSO_3_, 15.3% (C_4_H_4_O_6_)KNa·4H_2_O, and 0.38% phenol) was added [27]. The mixture was incubated in a boiling water bath for 5 min. After cooling, 3 mL of water was added and the absorbance was measured at 550 nm using an Infinite 200 PRO TECAN microplate spectrophotometer (Tecan, Austria). The background control was measured using the inactivated extract. The negative control of laminarin and CMC was incubated in the same conditions. The activity was expressed as µmol glucose/min/mg protein.

####  Guaiacol peroxidase (GPX) activity

The GPX activity was determined based on the transformation of guaiacol to tetra-guaiacol. The reaction was carried out with the use of 200 µL of extract sample, 350 µL of 100 mM phosphate buffer (pH 6.5), and 250 µL of 60 mM guaiacol (Sigma-Aldrich, USA). It was initiated by adding 200 µL of 0.25% H_2_O_2_. The absorbance was measured at 470 nm for 1 min using a Jasco V-730 spectrophotometer (Jasco, Hachioji-shi, Tokyo, Japan). The activity of the enzyme was calculated from the molarity coefficient ε = 26,6mM^− 1^cm^− 1^. The activity was expressed in µmol tetra-guaiacol/min/mg protein – U/mg protein [13, 25].

###  Molecular studies – western blotting & ELISA

####  Western blotting

The analyses of the fruits were performed using molecular techniques according to our previous work [28]. Approximately 5 g of fruit tissue was ground in liquid nitrogen, and then extraction buffer (65 mM Tris HCl, pH 6.8, 2% (w/v) SDS, 700 mM β-mercaptoethanol, 2 mM EDTA, 1:10 volume protease inhibitor and 1 mM PMSF) was added at a volume ratio of 1:1. The mixture was boiled at 99 °C for 5 min and centrifuged for 10 min at 4 °C. After Bradford assay quantification, the samples were separated by SDS-PAGE in a 12.5% resolving gel and a 4% stacking gel and then transferred to a PVDF membrane with a 0.2 μm pore size (Thermo Scientific, USA). The membrane was blocked with 5% BSA dissolved in TBST buffer for 1 h at room temperature. The incubation with primary antibodies was carried out at a concentration of 1:500 in 2.5% BSA in PBS for 2 h at room temperature. Next, the membrane was incubated for 2 h at room temperature with secondary antibodies conjugated with alkaline phosphatase (AP, Sigma, USA) at a concentration of 1:1000. After washing steps, the detection of bands was performed using BCiP and NBT according to the product protocol. Briefly, the AP substrates: 5-bromo-4-chloro-3-indolylphosphate (BCiP, Sigma, USA) in water (4 mg/mL) and nitro-blue tetrazolium (NBT, Sigma, USA) in water and N, N-dimethylformamide (DMF, Thermo Scientific, USA) (9 mg/0.3 mL water/ 0.7mL DMF) were used in the dark. The Pierce™ Prestained Protein MW Marker (Thermo Scientific, USA) was used for qualitative and quantitative analyses. The quantitative analyses of sample bands were performed using the GelDoc Go Imaging System (Bio-Rad, USA) with Image Lab Software v. 6.1 (Bio-Rad, USA). Three independent analyses were carried out.

####  ELISA

First, the samples were added to each well on a 96-well plate (Nunc MaxiSorpTM flat-bottom, Thermo Fisher Scientific, Denmark) and immobilized on a shaking plate at 37 °C for 72 h. Next, the washing step with 0.1% BSA in PBS at 37 °C for 1 h and incubation with primary antibodies at a concentration of 1:20 in PBS was carried out at 37 °C for 1 h. Then, the plate was incubated with the secondary antibody with AP in a dilution of 1:500 in PBS at 37 °C for 1 h. After the incubation and washing steps, the reaction was developed with a solution of p-nitrophenol phosphate (PNPP) according to the Thermo Scientific instructions. The reaction was stopped with 2 M NaOH after 20 min. The absorbance was measured in an ELISA reader (MPP-96 Photometer, Biosan) at 405 nm and analysed with Statistica v.13 tools (TIBCO Software Inc. USA). Analysis of variance (one-way ANOVA) and Tukey’s Honestly Significant Difference (HSD) post hoc test were used to compare the mean results. For all analyses, the significance level was estimated at *p* < 0.05.

##  Results

###  In situ studies on cell wall assembly

**Fig. 1:**
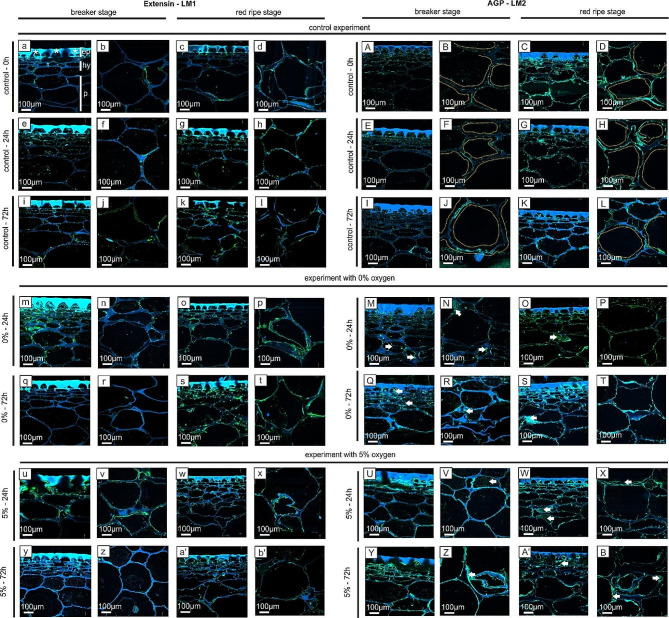
Distribution of extensin (a – b’) and arabinogalactan proteins (**A** – **B**’) in the fruits examined at the BR and RR stages after 24 h and 72 h of treatment with 0% O_2_, 5% O_2_ and 21% O_2_ (control). Photographs of sections of the external part of the fruits (epidermal and hypodermal layers) and their parenchymal part. The results were obtained using immunolabelling with the LM1 antibody recognizing extensin and the LM2 antibody recognizing AGPs. Double staining with Calcofluor White (blue color) was performed for better visualization of specific epitope localization (green color). Bars: 100 μm. Abbreviations: ep – epidermis, hy – hypodermis, p – parenchyma, * - cuticle. Indications: dot lines – specific AGPs localization, arrows – AGPs presence inside cells and in detached cellular compartments

**Fig. 2:**
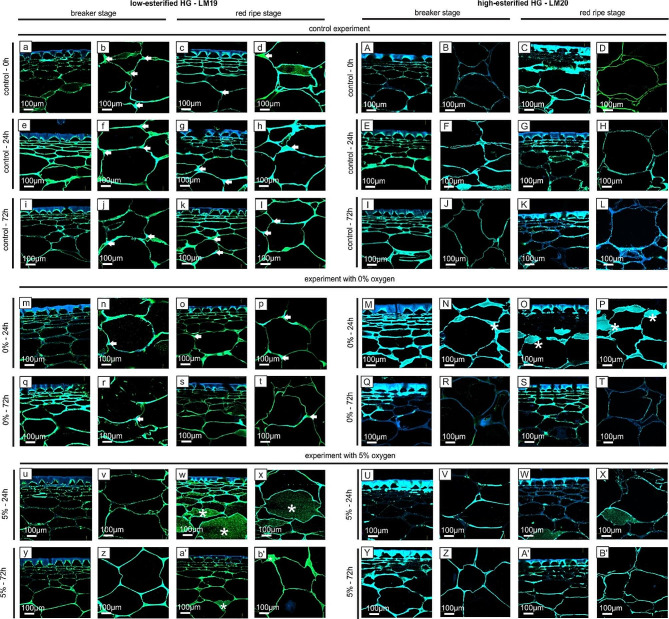
Distribution of low methyl-esterified homogalacturonan (a – b’) and high methyl-esterified homogalacturonan (**A** – **B**’) in the fruits examined at the BR and RR stages after 24 h and 72 h of treatment with 0% O_2_, 5% O_2_ and 21% O_2_ (control). Photographs of sections of the external part of the fruits (epidermal and hypodermal layers) and their parenchymal part. The results were obtained using immunolabelling with the LM19 antibody-recognizing low methyl-esterified HGs and the LM20 antibody-recognizing high methyl-esterified HGs. Double staining with Calcofluor White (blue color) was performed for better visualization of specific epitope localization (green color). Bars: 100 μm. Indications: arrows – specific localization in the tricellular junctions, asterisks – presence inside cells and/or in swollen cell walls

**Fig. 3:**
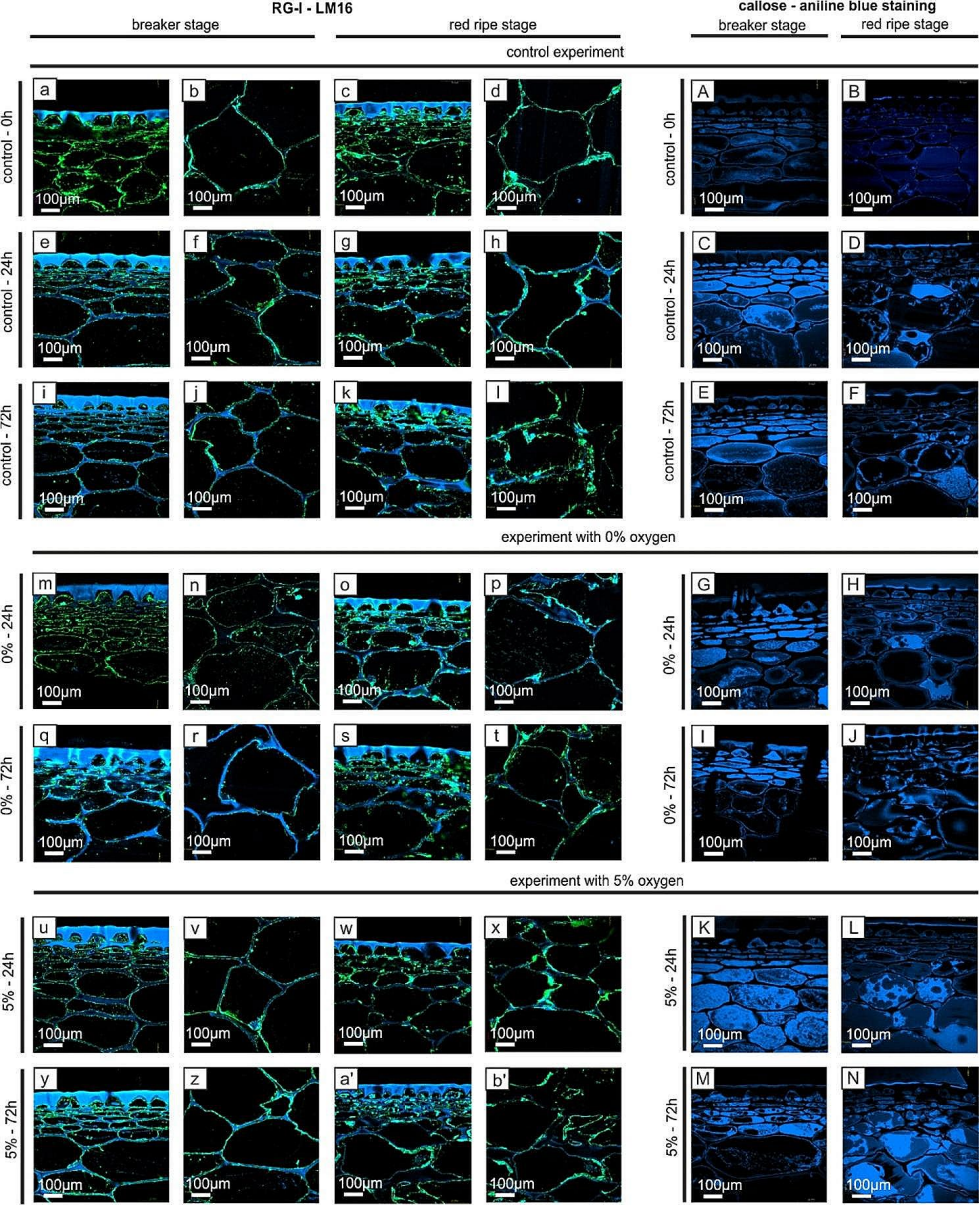
Distribution of rhamnogalacturonan-I (a – b’) and callose (**A** – **N**) in the fruits examined at the BR and RR stages after 24 h and 72 h of treatment with 0% O_2_, 5% O_2_ and 21% O_2_ (control). Photographs of sections of the external part of the fruits (epidermal and hypodermal layers) and their parenchymal part. The results were obtained using immunolabelling with the LM16 antibody recognizing RG-I. Double staining with Calcofluor White (blue color) was performed for better visualization of specific epitope localization (green color) (a – b’). Aniline blue staining was used for the detection of callose (**A** – **N**). Bars: 100 μm

The microscopic analyses of the cell wall assembly included immunofluorescence labelling of extensin (Fig. 1a – b’), arabinogalactan proteins (Fig. 1A – B’), low methyl-esterified homogalacturonan (Fig. 2a – b’), high methyl-esterified homogalacturonan (Fig. 2A – B’), and rhamnogalacturonan-I (Fig. 3a – b’). For this purpose, appropriate antibodies were used, to show that the oxygen stress caused changes in their distribution at the cellular level: in the external layer, which is composed of an epidermis (ep) covered by a cuticle (*) and a hypodermis (hy) as well as in parenchymal (p) tissues (Fig. 1a).

The in situ qualitative analysis on extensin (LM1 epitope) did not show significant differences between the control samples (Fig. 1a – l) and samples exposed to the low oxygen treatments, regardless of the duration of the experiment (Fig. 1m – b’). Also, the immunofluorescence indicating the presence of the LM1 epitope was very weak in the fruits at both stages. The examined LM1 epitopes were localized in similar cellular compartments in all samples, mainly at the border of the cell walls, both in external layers and in parenchyma cells. The observed extensin labelling pattern is typical for tomato fruit tissue. Here, as a result of low oxygen stress, no changes were noted in either the intensity of immunofluorescence or the localization of epitopes (Fig. 1a – b’).

Interestingly, the LM2 antibody recognizing arabinogalactan proteins revealed differences between the control samples (Fig. 1A – L) and the fruits exposed to the low oxygen treatment (Fig. 1M – B’). The mobility of AGPs allows them to migrate across the cell wall-plasma membrane continuum, and their localization is specified by a spatio-temporal pattern [19]. Here, the LM2 epitopes were also localized in the line at the border between the cell wall and the plasma membrane (Fig. 1B, D, F, H, J, L, dot lines). However, the low oxygen treatment in the experiments with 0% and 5% O_2_ caused a disruption of the wall’s continuity, and the AGP epitopes were found primarily in these damaged and detached cellular elements, also inside cells that were not attached to the cell wall surface (Fig. 1M – B’, arrows). Moreover, slightly higher immunofluorescence intensity was detected in the fruit sections in both stages after the low oxygen treatment.

The distribution of homogalacturonans (HGs) varied depending on the esterification degree (Fig. 2a – b’). LM19 recognizing low methyl-esterified homogalacturonan was characterized by the highest fluorescence intensity among all the antibodies used. The use of the LM19 antibody gave extremely distinctive labelling effects. Generally, LM19 epitopes were present in specific cellular areas, mainly in tricellular junctions and middle lamella (Fig. 2a – b’, arrows). In the case of fruit after treatment with 0% oxygen concentration, no significant changes in LM19 epitope arrangement were observed compared to control fruit (Fig. 2m – t). Interestingly, in the fruit at the RR stage of the experiment with 5% oxygen, the immunolabelling was observed even in the spaces inside the cells (Fig. 2w, x, a’, asterisks).

The labeling with the LM20 recognizing high methyl-esterified homogalacturonan (Fig. 2A – B’) was different than in the case of labeling with the LM19 antibody. Firstly, the LM20 epitopes were distributed in the whole area of the cell walls, filling the entire space of the cell wall, without a specific distribution pattern. Secondly, the presence of LM20 epitopes in the fruits exposed to the low oxygen treatment was significantly lower than in the control samples. The decreased presence of LM20 epitopes is emphasized by the blue-color fluorescence visible in the cell wall surfaces as the result of the Calcofluor blue double staining (Fig. 2M – B’). The exception is the sample after 24 h of treatment with 0% oxygen, where accumulated epitopes were noted in the changed, swollen cell walls (Fig. 2M – P, asterisks). In other cases, in the storage atmosphere with 5% oxygen, a low fluorescence signal was observed, regardless of the duration of the fruit treatment (Fig. 2U – B’).

The labelling of rhamnogalacturonan-I using the LM16 antibody showed no differences in its distribution in the fruits in all the examined samples (Fig. 3a – b’). The stages of ripening, the duration of the experiment, and the low oxygen treatment had no influence on the RG-I localization in the fruit cells. The distribution of RG-I was very unspecific, and the epitopes were noted in all spaces of the cell walls.

Interestingly, the results obtained using Aniline blue staining showed an increase in callose secretion in individual samples (Fig. 3A – N). At the start of the experiment (0 h), no callose was observed in the fruits in both stages of ripening. In turn, after 24 h and 72 h of storage, callose secretion was observed in the control fruits, mainly at the BR stage. After the low oxygen treatments, an increase in the callose content in the cells was visible. The fluorescence signal was more intensive in the fruits at 5% oxygen, where callose was visualized in the whole external layers of the fruits.

###  Enzymatic activity

**Fig. 4:**
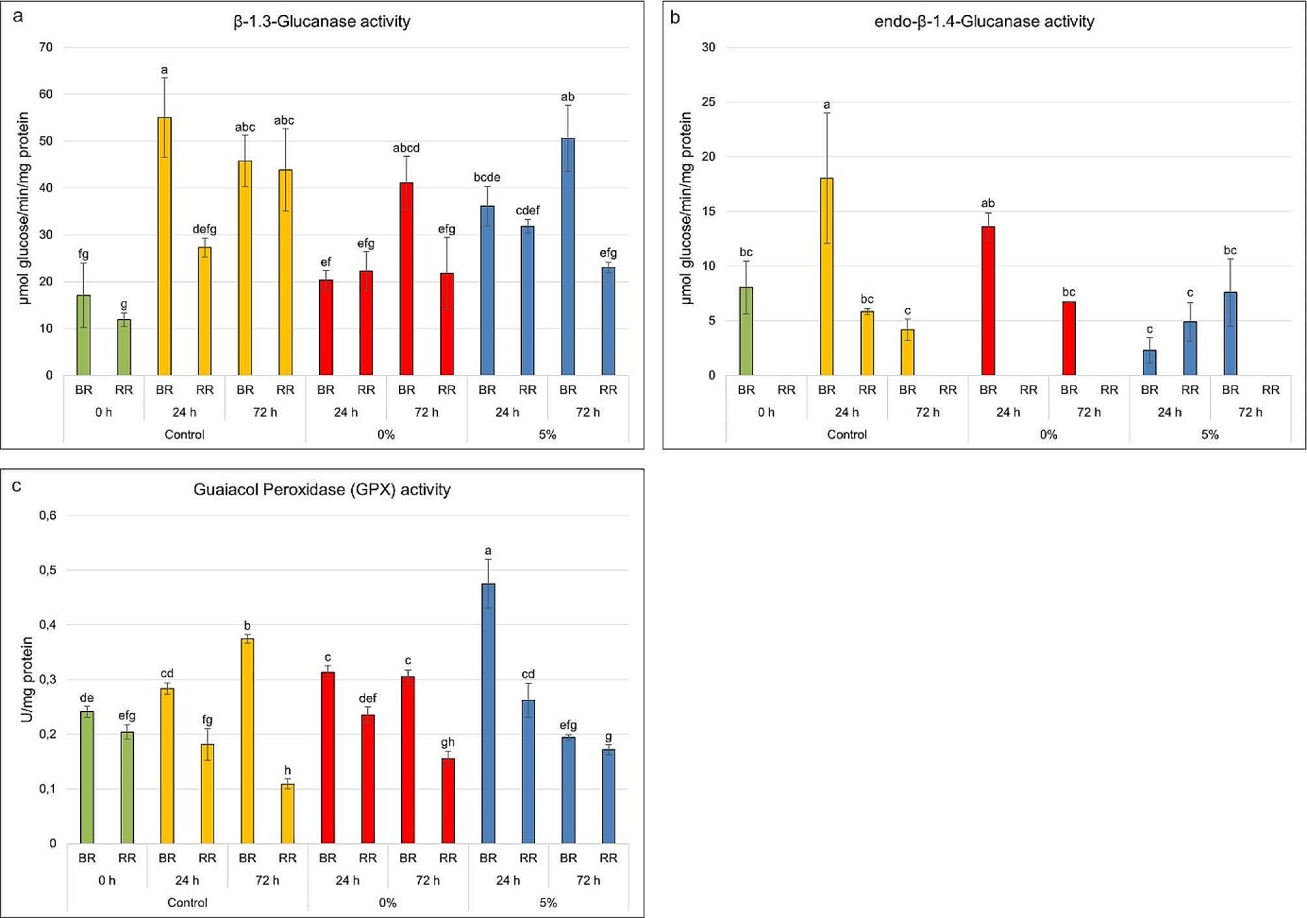
Activities of β-1,3-glucanase (**a**), endo-β-1,4-glucanase (**b**), and guaiacol peroxidase (GPX) (**c**) in the fruits examined at the BR and RR stages after 24 h and 72 h of treatment with 0% O_2_, 5% O_2_ and 21% O_2_ (control). Statistical tests: One-way ANOVA, Tukey HSD, *p* < 0.05. Bars show the standard error. Different letters indicate significant differences in the enzyme activity

The activities of β-1,3-glucanase, endo-β-1,4-glucanase, and guaiacol peroxidase (GPX) are presented in Fig. 4a – c. The initial β-1,3-glucanase activity was 17 µmol glucose/min/mg protein and 11 µmol glucose/min/mg protein in the fruits collected (0 h) at the BR stage and at the RR stage, respectively. The greatest and statistically significant increase in the activity of this enzyme was observed in the control fruit samples, with the activity reaching 55 µmol glucose/min/mg protein after 24 h at the BR stage and decreasing to 45 µmol glucose/min/mg protein after 72 h in both the BR and RR stages. At 0% oxygen, only the fruits at the BR stage showed a significant increase in enzyme activity after 72 h compared to the fruits at time 0. In the same conditions, the activity of this enzyme in the fruits at the RR stage was 2-fold lower than that in the samples stored in the control conditions for 72 h. The storage of the fruits at 5% oxygen also reduced the activity of this enzyme compared to the control conditions, but to a lesser extent than the low oxygen conditions. After 24 h, an increase in β-1,3-glucanase activity was observed in the fruits at the BR stage compared to the samples at time 0 (36 µmol glucose/min/mg protein), but it was lower than that in the fruits kept in the control conditions. As in the experiment with 0% oxygen, the activity of this enzyme in the fruits at the BR stage increased after 72 h to a level of 50 µmol glucose/min/mg protein (Fig. 4a).

Endo-β-1,4-glucanase activity was observed mainly in the fruits at the BR stage. At the RR stage, the activity of this enzyme was only observed in the fruits stored in the control conditions and with 5% oxygen access at a glucose rate of 5 µmol/min/mg protein. The highest increase in the endo-β-1,4-glucanase activity was observed in the control conditions at 18 µmol glucose/min/mg protein and in the 0% oxygen environment at 13 µmol glucose/min/mg protein. In both cases, a decrease in endo-β-1,4-glucanase activity to the level observed in the fruits at time 0 was recorded after 72 h. The storage at 5% oxygen availability had the highest effect on the activity of this enzyme. After 24 h, a sharp decrease to 2 µmol glucose/min/mg protein was observed in this parameter, which then increased to 7.5 µmol glucose/min/mg protein, approaching the activity in the control fruits (Fig. 4b).

The initial (0 h) guaiacol peroxidase (GPX) activity in the fruits at both ripening stages (BR and RR) was similar, i.e. 0.2–0.24 U/mg protein. The activity of GPX in the control samples increased significantly at the BR stage after 24 h and 72 h compared to the sample at time 0. In turn, a decrease in the activity of this enzyme was observed at the RR stage. A similar pattern of GPX activity was observed in the 0% oxygen conditions during fruit storage. Only after 72 h in these conditions in the BR stage was the activity at the same level as after 24 h at 0% oxygen and in the control - ~0.30 U/mg protein. The 5% oxygen content had the greatest effect on GPX activity in the fruits. After 24 h, a 50% increase in the activity of this enzyme was observed in the fruits at the BR stage, which then dropped sharply after 72 h to a level of 0.19 U/mg protein. The oxygen deficiency in the fruits at the RR stage inhibited the decrease in GPX activity after both 24 h and 72 h compared to the fruits kept in the control conditions (Fig. 4c).

###  Molecular characteristics: qualitative and quantitative analyses of cell wall composition

**Fig. 5:**
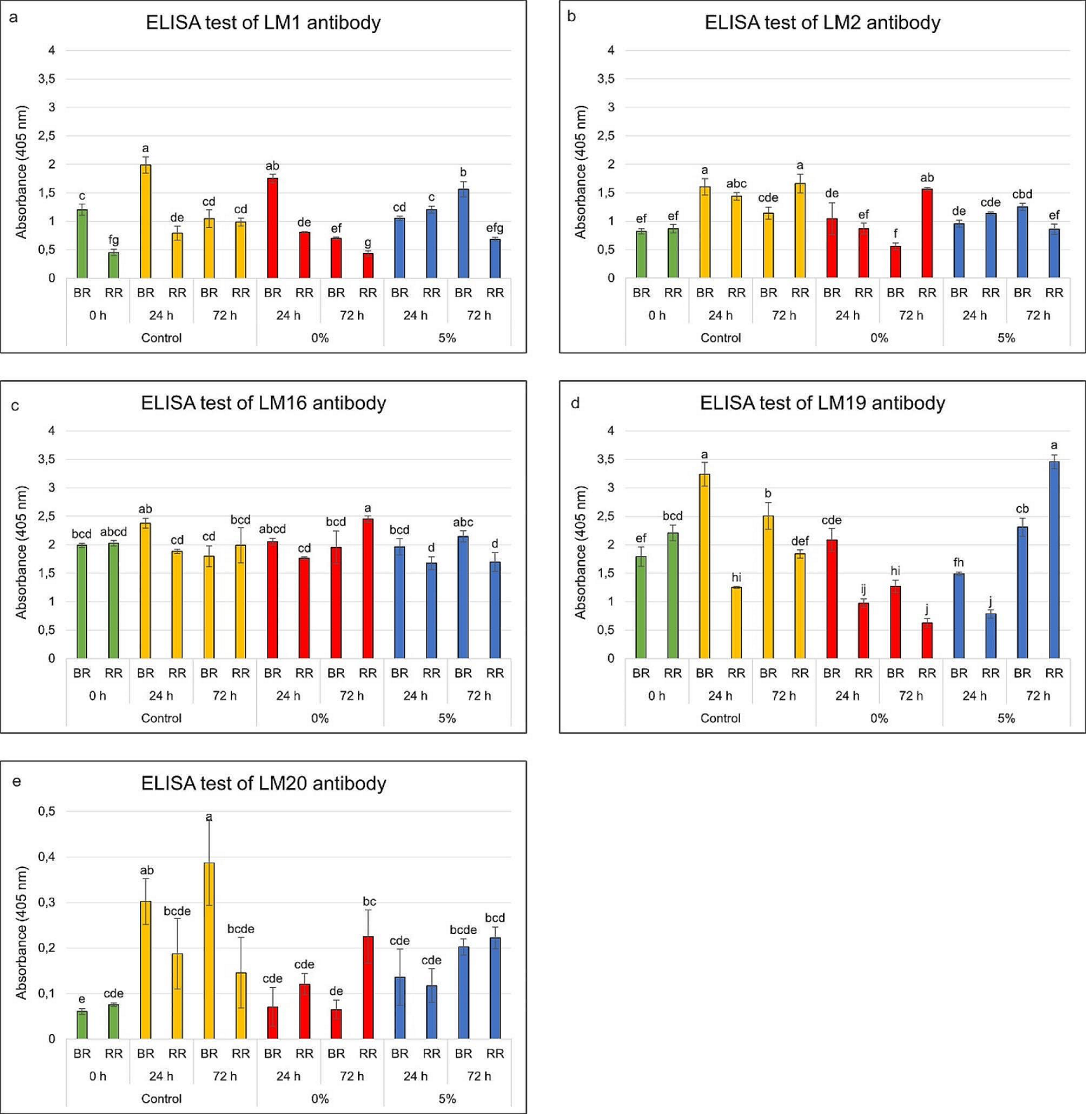
Quantitative analyses of extensin (**a**), arabinogalactan protein (**b**), rhamnogalacturonan-I (**c**), low methyl-esterified homogalacturonan (**d**), and high methyl-esterified homogalacturonan (**e**) content in the fruits examined at the BR and RR stages after 24 h and 72 h of treatment with 0% O_2_, 5% O_2_ and 21% O_2_ (control). The results were obtained using an ELISA test with LM1, LM2, LM16, LM19, and LM20 antibodies. Statistical tests: One-way ANOVA, Tukey HSD, *p* < 0.05. Different letters indicate significant differences among the examined samples

The qualitative results were verified by another technique using the same antibodies, i.e. ELISA (Fig. 5a – e). The initial level (0 h) of the LM1 epitope in the fruits at the BR stage was nearly three times higher than in the fruits at the RR stage. The storage in the control conditions induced a decrease in their content with the progress of ripening. The experiment with 0% O_2_ did not affect the presence of extensin in the fruit and their quantitative changes proceeded as in the control experiment. A slight alternation represents a decrease in the quantity of LM1 in fruit at the RR stage after 72 h of treatment with 0% oxygen (Fig. 5a). In the case of the experiment with 5% O_2_, no downward trend in its content was observed after the 24 h treatment. However, the longer exposure in these conditions resulted in a significant decrease in the extensin content in ripe fruit (Fig. 5a).

AGPs recognized by LM2 antibody were present in the examined fruit in similar amounts (Fig. 5b). The AGP content increased in the control samples at 24 h after fruit harvest, while remaining at relatively constant levels. In fruit stored in conditions of 0% and 5% oxygen concentration, the amount of AGP remained at a similar level after 24 h. A reversal of the trend was observed in fruits at the RR stage, in which after 72 h of storage in 0% oxygen the AGP content increased and in 5% oxygen it slightly decreased (Fig. 5b).

In the case of the LM16 epitopes, no significant differences were noted (Fig. 5c). Interestingly, the experimental conditions caused the greatest changes in the HG content (Fig. 5d and e). First of all, the level of absorbance for both antibodies is completely different. While in some samples the absorbance measured for LM20 was impossible to detect, in the case of LM19 absorbance value was several dozen times higher. The 1-day and 3-day fruit storage in the control conditions resulted in a significant increase in homogalacturonan content mainly at the BR stage (Fig. 5d and e). Briefly, the increase in its content was not as substantial in the fruit kept in the low oxygen conditions. In the fruit at the BR stage stored for 24 h, the content of the low methyl-esterified homogalacturonan (LM19 epitope) was clearly higher in comparison to its initial content. But the treatment with 0% O_2_ resulted in reduced content in the fruit at both stages. In turn, the treatment with 5% O_2_ for 72 h induced a significant increase in its presence (Fig. 5d). What is important, a completely different trend was observed in the case of high methyl-esterified homogalacturonan (Fig. 5e). Firstly, a significant decrease in the content of LM20 epitopes in fruit exposed to low O_2_ conditions was noted, compared to the control experiment. In turn, a slight increase in their quantity in the fruit persisted for 72 h, indicating a clear disorder in the HG degradation process (Fig. 5e).

**Fig. 6:**
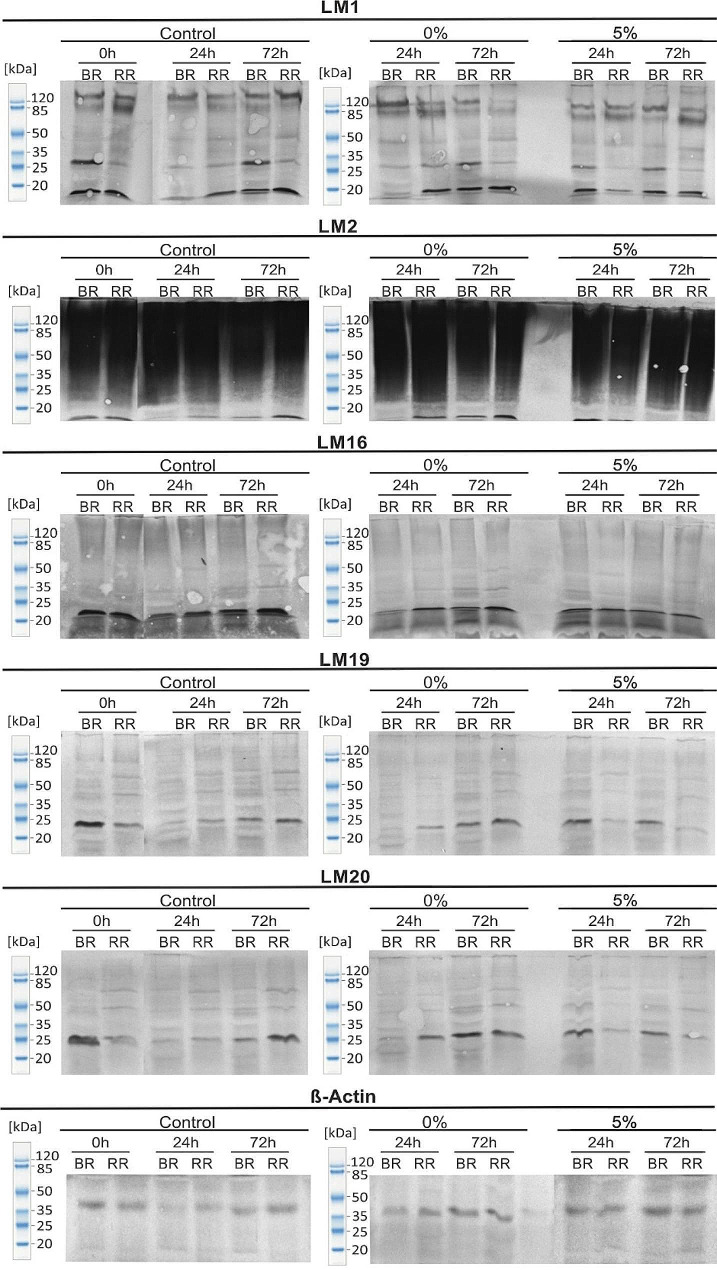
Molecular analyses of extensin, arabinogalactan protein, rhamnogalacturonan-I, low methyl-esterified homogalacturonan and high methyl-esterified homogalacturonan in the fruits examined at the BR and RR stages after 24 h and 72 h of treatment with 0% O_2_, 5% O_2_ and 21% O_2_ (control). The results were obtained using Western blot analyses with LM1, LM2, LM16, LM19, and LM20 antibodies. β-Actin used as a loading control. Molecular mass (kDa) is indicated on the left side of the photograph

The quantitative changes were correlated with molecular modifications of particular cell wall components. Our goal was to use this method to assess the levels of tested ingredients and provide a visual representation of their degradation over time. Unfortunately, the changes in molecular mass between samples were very small and very difficult to determine (Fig. 6). Western blotting with the LM1 antibody revealed degradation of extensin in the 0% oxygen conditions after 72 h. Thus, there was a diminishing of bands at 85–120 kDa which might be associated with response to experimental conditions. Also, the presence of RG-I with a low molecular mass, even lower than 20 kDa has been detected. The appearance of strong, intense bands of low molecular weight RG-I in all samples indicates that RG-I is significantly degraded and its degradation can be associated with various physiological processes, such as stress response or cell wall remodeling.

On the contrary, the detection with the LM2 antibody visible as smear bands along the whole lanes indicated the most frequent presence of the AGP epitope with a molecular weight in the range of 20 kDa-120 kDa. Smearing of these bands is typical for AGPs in fruit recognized by LM2, where the bands appear diffuse or streaky. Results with the LM2 antibody did not show differences between samples but exhibited the strongest reactivity against the AGP. Visible strong detection was noted in the case of the fruits from the low oxygen treatment and the control experiment.

The detection of the LM19 and LM20 epitopes indicated a very large range in the molecular weights of the HG molecules. Besides the fewer bands with a higher molecular size, the bands indicating the presence of HGs with low molecular weights (~ 30 kDa) were the most intense (Fig. 6). The detection of HGs appearing in the entire band indicates the ongoing degradation process of HG. This phenomenon indicates partial breakdown of the polysaccharide, resulting in fragments of varying sizes rather than a single, distinct band. Here we noted that the degradation in the cell wall during fruit ripening is a complex and dynamic process involving modification of all cell wall components.

##  Discussion

Benkeblia [29] reviewed the beneficial effect of hypoxia in fresh crops, e.g. reduction of physiological disorders, browning, internal breakdowns, and deastringency. Analyses on peach fruit response to anoxia showed that ripening was delayed in treated fruits, which was accompanied by prevention of fruit softening, colour changes, and ethylene production [30]. Moreover, the induction of fermentative pathways, glycolysis and changes in enzymatic activities involved in sucrose synthesis and degradation were induced by anoxia [30]. The action of low oxygen on fruit ripening involves suppression of *de novo* synthesized ripening proteins and mRNA transcripts [11]. Furthermore, hypoxia also modulates posttranscriptional and posttranslational processes occurring in fruit cells [31]. Additionally, storage in low oxygen environments affects the production of volatiles, cell wall-related metabolism, and energy-related metabolism [5, 11, 32, 33]. The most important quality parameter and the subsequent effect of the low oxygen storage on fruits is the maintenance of fruit firmness [34]. Thus, the purpose of a study conducted by Mojević and Tešanović [34] was to define the physical, chemical, and sensory features of tomato fruits at mature green and pink stages kept in anoxia conditions for 24 h. Firstly, control matured green and pink fruits showed higher weight loss than anoxia-treated samples. Also, the anoxia-treated mature green fruits exhibited higher firmness values than the control fruits. In turn, fruits at the pink stage treated with anoxia showed a lower firmness value [34]. It is well known that the loss of firmness is closely related to modifications in the architecture of cell wall components [35]. However, the mechanisms that influence these changes in fruits have still not been elucidated. In view of the available information and the limited experimental data on the mechanism of action of low oxygen in fruit ripening, the present paper describes molecular studies on the cell wall composition in fruits stored in a low oxygen environment.

The results of the current study indicate crucial changes in the assembly of the cell wall in response to the experimental conditions. Carried out molecular analysis helped to explain why fruits stored under anoxia and hypoxia oxygen stress varied from the control samples. Firstly, the microscopic studies showed numerous deviations from the typical distribution pattern of the cell wall assembly. In general, the greatest difference was observed in the localization of pectins and, above all, low methyl-esterified HGs. In fruit stored in conditions of 5% oxygen concentration, HGs next to the middle lamella and tricellular junctions filled also the intracellular spaces. This is even more interesting because the analysis of fluorescence intensity in fruit in experimental conditions indicates unchanged or slightly lower content of low- and high-esterified HGs, compared to control conditions. Also, the disrupted distribution of high-esterified HGs, localized in swollen cell walls, was observed in the fruit stored for 24 h in 0% oxygen. In the case of the RG-I analysed, the changes were not so spectacular. Another effect observed in situ is the localization of AGPs and their increased presence in detached cellular compartments. It is known that AGPs are proteins with a very specific pattern of occurrence in the cell, hence it was easy to note modifications in their arrangement as a result of changed oxygen conditions, both in 0% as well as in 5% oxygen concentrations. The wrecked arrangement of the components disrupts the structural interactions in the cell assembly. Disturbed spatio-temporal distribution of cell wall compounds indicates changes in the structure of the cell walls, which undoubtedly affect the condition of the fruit.

Secondly, the molecular analyses confirmed the results obtained in situ, clearly indicating differences in the content of individual components in the cells of control fruits and those stored under oxygen stress conditions. The content of HGs with different levels of esterification was clearly changed in the fruit stored in low oxygen conditions. It is worth emphasizing the significantly lower amount of low methyl-esterified homogalacturonan in the fruit stored in 0% oxygen for 24 and 72 h. In the case of 72 h of exposure, the content decrease is twofold, both in the BR and RR stages. The effect of 5% concentration is different, 24-hour storage causes a similar decrease in LM19 epitope content, but longer storage for 72 h causes its significant increase. We assumed that in the 5% oxygen experiment, a clearly lower rate of HG degradation in the fruit was observed at the RR stage, which was visible only after longer treatment. This unquestionably indicates a beneficial effect of the 5% oxygen treatment on the ripening process and on the rate of its progress. Conditions of both 0% and 5% oxygen concentration resulted in a decrease in the content of the LM20 epitope after 24 h of exposure. The level of methyl-esterified homogalacturonan was lower in fruit treated with 0% oxygen for 24 h than in the control fruit. However, 72-hour exposure resulted in an increase in their presence in fruit at the RR stage. This state also persisted in storage at 5% oxygen and the level was still lower than under controlled conditions. The 72-hour exposure slightly increased their presence compared to 24 h, which also confirms that only a longer treatment produces the potential effect. Our research goes hand in hand with previous reports in which in apple fruit, the content of total pectin and hemicellulose was higher at 3% oxygen compared to a 1% oxygen environment [36, 37]. These changes were driven by numerous enzymes leading to the solubilization and depolymerization of pectins and hemicellulose [36]. Furthermore, it is well known that demethylation allows pectin to interact with calcium, resulting in the strengthening of the cell wall and middle lamellae [38]. It is also interesting that not all pectin compounds react to experimental conditions at the same level, i.e. very small impact on the RG-I appearance. On the basis of the obtained results, it can be hypothesized that the fruit changes in the anoxic and hypoxic environments were mainly associated with a modified content and distribution of low methyl-esterified HGs. Taking into account that this type of pectin has the ability to bind calcium ions, we conclude that this is related to maintaining the continuity of the cell wall under oxygen stress conditions. The increased HG content as a response and defence mechanism of the fruit cell may be related to the strengthening and protection of the stressed tissue.

Studies on pears carried out by Pedreschi and coworkers [39] have demonstrated that, in order to surpass stressful conditions, the primary metabolic pathways are altered, e.g. protein synthesis during storage in a controlled atmosphere. In their work, the protein profiles have indicated that proteins are down-regulated by low oxygen or high carbon dioxide concentrations. It was clarified that, despite the slight changes in the protein content, this may be highly important and correlated to their activity which, in turn, is related to defensive mechanisms [39]. Also, in studies described by Fragkostefanakis and coworkers [40], the transcription of a specific arabinogalactan protein was induced in tomato fruits by several stressors, including anoxia and hypoxia. The authors conclude that low oxygen conditions, mainly anoxia, may regulate fruit ripening by modulating the expression of specific cell wall-related genes [40]. In subsequent work, Western blot analysis showed that one type of apple protein (MdRAP2.12) was divergently accumulated in normoxic and hypoxic conditions. The highest level was reached in a 0.4 kPa oxygen environment. In our study, the analysis of protein content using specific techniques with LM1 and LM2 antibodies showed rather slight disturbances in protein metabolism as a response to the experimental conditions. Data obtained on arabinogalactan proteins and extensin indicate that low oxygen stress has an impact on their occurrence in cell walls but the changes are very small in comparison to the noted HGs alterations. The 0% oxygen conditions result in a decrease in AGP content after 24 h of treatment. A clear degradation of AGPs is also present in fruit at the BR stage after 72 h, but already in fruit at the RR stage, the AGP content is comparable to the control fruit. These data have highlighted that mature apple tissues specifically modulate sensing and regulatory mechanisms in response to hypoxic stress conditions [31]. Storage of fruit in 5% oxygen concentration also influenced the presence of AGPs in fruit at both ripening stages. Compared to fruit from the control experiment, those after 24 h and 72 h exposures contained a lower amount of AGPs.

In previous studies, Pedreschi and coworkers [39] concluded that fruit cells adapt to stress conditions (under browning-inducing and varying O_2_ concentrations) by up-regulation of the key enzyme triosephosphate [39]. Also, the unquestionable involvement of enzymes in the process of fruit softening has been reported [41]. In the current study, the activities of β-1,3-glucanase, endo-β-1,4-glucanase, and guaiacol peroxidase were analysed. The selected enzymes are related to fruit cell metabolism and, more precisely, to the secretion of callose and the process of fruit ripening [12, 42]. In a study carried out by Choudhury and coworkers [12], changes in the enzymatic activity of β-1,3-glucanase in association with the pattern of alterations during fruit ripening were investigated. In bananas, low glucanase activity was detected during the early stages of ripening at 1–5 days after harvest. Then, glucanase activity was slowly enhanced during 3–5 days after harvest. Next, it increased steadily during the subsequent stages of ripening and then gradually decreased towards the full ripe stage. Detailed results indicate that the substrate for glucanase is mostly present in the unripe fruit and disappears in the ripe fruit [12]. Similar activity fluctuations were noted in our work on tomato fruits. In the fruits after harvest, β-1,4-glucanase activity was noted only at the BR stage. The 24-h storage in the control conditions resulted in the appearance of its activity also in the RR stage. However, the longer experiments (72 h) showed that glucanase activity decreased in the fruits at the RR stage. Interestingly, the low oxygen treatment exerted an effect on the examined activity. Indeed, glucanase activity was also present in the fruits at the RR stage kept in 5% oxygen after 24 h. In turn, the anoxic conditions induced a gradual decrease in glucanase activity after 24 h and 72 h of the experiment. It can be assumed that glucanase activity is a sensor of progressing to the ripening process. Taken together, it may be confirmed that the 5% oxygen concentration may slow down the ripening process and 0% oxygen can accelerate the changes taking place during ripening. Moreover, the analyses of guaiacol peroxidase activity, i.e. an antioxidant enzyme [43], proved that such stress factors as reduced oxygen content can change its activity. This trend of changes was similar in the control sample and in the fruits after the treatment with 0% oxygen. The guaiacol peroxidase activity increased in the fruits at the BR stage and decreased in the fruits at the RR stage. Interestingly, in the fruits at the BR stage kept in the 5% oxygen environment, guaiacol peroxidase activity significantly increased and rapidly decreased after 24 h. These results indicate that a hypoxic and anoxic environment directly impacts ripening progress.

Another interesting topic addressed in our current research was callose detection. It has been proved in many scientific reports that callose, i.e. β-1,3-glucan, is a polymer secreted as a result of emerging stress conditions. Moreover, callose inhibits the invasion and spread of pathogens in fruits during postharvest storage [20]. and controls intercellular transport, including signalling, through cell wall channels [44]. The importance of glucanases in plant adaptation towards environmental cues also included β-1,3-glucanases activity in the process of accumulation and degradation of callose [41, 42]. An analysis of microarray data revealed different expression patterns of β-1,3-glucanase genes in particular stages of fruit development [41, 42] and ripening [44]. Increased secretion of callose was also observed in the tomato fruits analysed in the present study. Callose was not present in the microscopic sections of freshly picked fruits. In turn, β-1,3-glucan was very abundant in the stored fruits and in the fruits treated with the low concentrations of oxygen. In addition, the β-1,3-glucanase activity correlated with the presence of callose, whose levels rapidly increased in the fruits after harvest. This can be considered a way of remodelling the cell wall in response to unfavourable conditions. The enhanced local accumulation of callose may play protective functions and be part of the defence strategy of fruits against low oxygen stress. Nevertheless, in contrast to other well-studied cell wall substances, such as pectins, the detailed mechanism by which callose may regulate fruit response to oxygen stress requires further investigation.

To sum up, the experiments carried out should result in specific information and a one-way indication of what conditions and how long their exposure affects the fruit, and at which stage of the ripening process the fruit is more susceptible to them. However, the significant number of deviations noted both in the in situ presence and molecular parameters of individual cell wall components make it difficult to draw clear conclusions. However, it is certain that low oxygen conditions change the structural features of the cell wall both at the beginning and at the end of the ripening process. In our assessment, less significant changes were observed in fruit in the BR stage than in the case of the RR stage. In riped fruit intriguing increases in HG content were observed, indicating an influence of oxygen stress on the progress of the ripening process. The duration of the experiment also influenced the observed changes. The first symptoms of changes were noted after 24 h, but only after 72 h, more significant deviations from the control level were visible. A clearer situation concerns the level of oxygen concentration. The 0% oxygen conditions generally reduce the content of cellular components, and the mentioned changes were more rapid. The 5% oxygen concentration causes a lower decrease in the ingredients content which increases after 72 h of exposure. The observed molecular reset occurring in tomato cell walls in hypoxic and anoxic conditions seems to be a result of regulatory and protective mechanisms modulating ripening processes. Additionally, it can be regarded as a beneficial effect in the context of fruit postharvest storage.

##  Conclusion

In the current work, an experiment using two oxygen concentrations: 0% and 5% was carried out. Its effect on fruit at the beginning and the end of the ripening process was examined. The influence of exposure times on given oxygen stress conditions was also investigated.

The results obtained elucidate the impact of low oxygen concentrations on fruit at the molecular level:

1. The in situ distribution of particular cell wall components has been changed as a result of low oxygen treatment – disorders in specific spatio-temporal patterns typical for AGPs and pectins during the ripening process.
2. Changes in molecular parameters of AGPs, pectins, and extensin are the result of the influence of changes in oxygen conditions - intensified or inhibited degradation processes occurring during fruit ripening.
3. The presence or absence of single constituents may affect the general arrangement of the cell wall as a network with a detailed organized assembly and specific interactions between components.
4. The obtained data confirm that oxygen deficiencies influence the ripening process, and the cell wall is associated with mechanisms supporting their possible protection against stress conditions.

### Electronic supplementary material

Below is the link to the electronic supplementary material.

Supplementary Material 1

## Data Availability

The datasets analysed during the current study are available from the corresponding author on reasonable request.
