# Supplementary material for: Low oxygen environment effect on the tomato cell wall composition during the fruit ripening process
Source: BMC Plant Biol. 2024 Jun 6;24:503. doi: 10.1186/s12870-024-05226-x (PMC11155102; doi:10.1186/s12870-024-05226-x)

**Supplementary materials:**

**Title:**

Low oxygen environment effect on the tomato cell wall composition during the fruit ripening process

**Authors:**

Agata Leszczuk, Nataliia Kutyrieva-Nowak, Artur Nowak, Artur Nosalewicz, Artur Zdunek

**Raw data for Figure 8.** Molecular analyses of extensin, arabinogalactan protein, low methyl-esterified homogalacturonan, high methyl-esterified homogalacturonan, and rhamnogalacturonan-I in the fruits examined at the BR and RR stages after 24h and 72h of treatment with 0%, 5% O_2_ and 21% (control) as well as at the start of the experiment (start).

The results were obtained using Western blot analyses with LM1, LM2, LM16, LM19, and LM20 antibodies. Molecular mass (kDa) is indicated on the left side of the photograph.


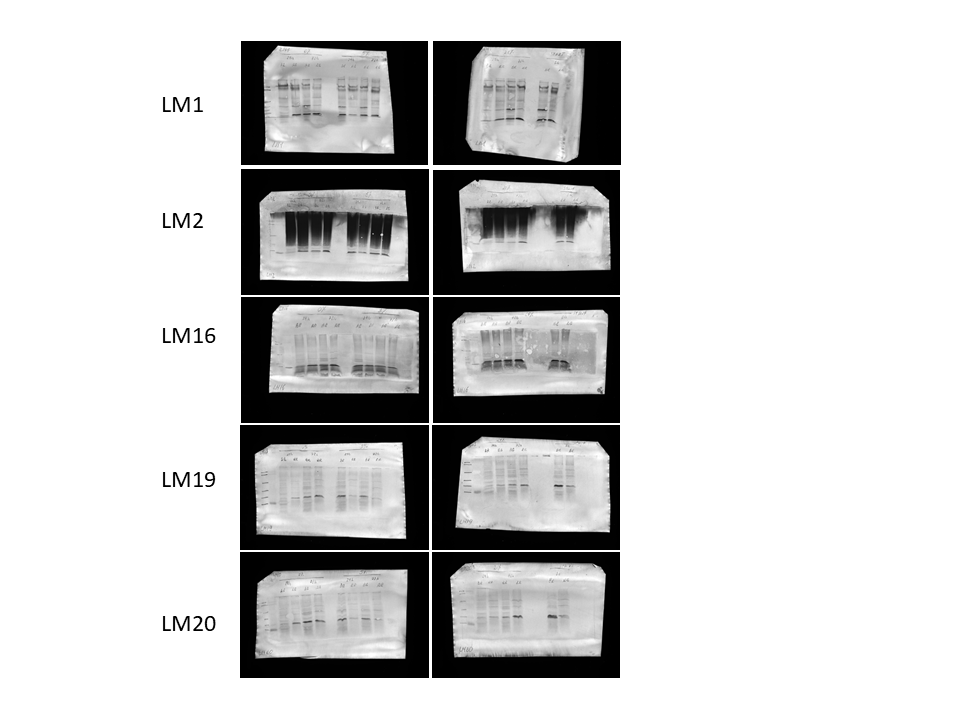

Supplement: Supplementary file 1 — Supplementary Material 1 [file 12870_2024_5226_MOESM1_ESM.docx]
